# Highly Efficient and Heritable Targeted Mutagenesis in Wheat via the *Agrobacterium* *tumefaciens*-Mediated CRISPR/Cas9 System

**DOI:** 10.3390/ijms20174257

**Published:** 2019-08-30

**Authors:** Shujuan Zhang, Rongzhi Zhang, Jie Gao, Tiantian Gu, Guoqi Song, Wei Li, Dandan Li, Yulian Li, Genying Li

**Affiliations:** 1 Crop Research Institute, Shandong Academy of Agricultural Sciences, Jinan 250100, Shandong, China; 2 Key Laboratory of Wheat Biology and Genetic Improvement on North Yellow and Huai River Valley, Ministry of Agriculture, Jinan 250100, Shandong, China; 3 National Engineering Laboratory for Wheat and Maize, Jinan 250100, Shandong, China

**Keywords:** *Agrobacterium tumefaciens*, CRISPR/Cas9, heritable, later generations, mutation, wheat

## Abstract

The CRISPR/Cas9 system has been successfully used in hexaploid wheat. Although it has been reported that the induced mutations can be passed to the next generation, gene editing and transmission patterns in later generations still need to be studied. In this study, we demonstrated that the CRISPR/Cas9 system could achieve efficient mutagenesis in five wheat genes via *Agrobacterium*-mediated transformation of an sgRNA targeting the D genome, an sgRNA targeting both the A and B homologues and three tri-genome guides targeting the editing of all three homologues. High mutation rates and putative homozygous or biallelic mutations were observed in the T0 plants. The targeted mutations could be stably inherited by the next generation, and the editing efficiency of each mutant line increased significantly across generations. The editing types and inheritance of targeted mutagenesis were similar, which were not related to the targeted subgenome number. The presence of Cas9/sgRNA could cause new mutations in subsequent generations, while mutated lines without Cas9/sgRNA could retain the mutation type. Additionally, off-target mutations were not found in sequences that were highly homologous to the selected sgRNA sequences. Overall, the results suggested that CRISPR/Cas9-induced gene editing via *Agrobacterium*-mediated transformation plays important roles in wheat genome engineering.

## 1. Introduction

The clustered regularly interspaced short palindromic repeats (CRISPR)/Cas9 system is becoming a powerful genome editing tool and has rapidly replaced the zinc finger nucleases (ZFN) and transcription activator-like effector nuclease (TALEN) systems. The CRISPR/Cas9 system can produce double-stranded breaks (DSB) at specific chromosomal sites that are subsequently repaired by error-prone non-homologous end joining (NHEJ) or the homology-directed repair (HDR) pathway. This approach is more convenient and powerful than random mutagenesis for inducing fixed-point mutations, thus presents many advantages for functional genomics and molecular breeding [1,2,3,4].

The CRISPR/Cas9 system for gene editing has been successfully used and developed in various plant species, and stable genetic mutants have been obtained [3,5,6,7,8,9,10,11,12,13,14,15]. However, the editing efficiency of different CRISPR/Cas9 systems can differ in different species even within the same species. Many factors may affect the efficiency of the CRISPR/Cas9 system, such as the *Cas9* gene, RNA polymerase III-dependent promoters (U3, U6, etc.), target sites, plant species (genome size, GC content), and transformation strategies involved [3,4,7,8]. For genome editing in plants, vectors carrying the Cas9 and sgRNA expression cassettes are delivered into plant cells via two major platforms: *Agrobacterium*-mediated and biolistic (particle bombardment) delivery systems. The *Agrobacterium*-mediated method presents the advantages of integrating T-DNA into plant genomes with a low copy number of transgenes, stability and heritability in progeny.

Common wheat (*Triticum aestivum*. L, 2n = 6x = 42, AABBDD) presents a large genome size of 17 Gb with a complex allohexaploid nature that consists of three different genomes (AA, BB and DD) [16,17]. Each genome is equivalent to that of a diploid plant, in which editing can result in homozygous, biallelic, heterozygous or chimaeric mutants. Compared to diploid plants, when targeting polyploid wheat, mutation detection and the analysis of editing results are much more challenging. The downstream analysis of the inheritance of different editing types is more difficult due to the complex hexaploid nature of the wheat genome. It has been demonstrated that the CIRSPR/Cas9 system can edit the genome of wheat protoplasts [18,19,20]. CRISPR/Cas9 technology has been used to generate targeted mutations in the mildew resistance locus *TaMLO-A1* allele in T0 transgenic plants [13]. Liang et al. [21] applied DNA-free genome editing of bread wheat using CRISPR/Cas9 ribonucleoprotein complexes (RNPs) to obtain completely transgene-free mutants. Sánchez-León et al. [22] generated low-gluten, non-transgenic wheat lines using CRISPR/Cas9, and these plants showed a large reduction in α-gliadins. Zhang et al. [23] used the gene-editing mutants of three homologues (*TaGW2-A1*, *-B1* and *-D1*) to analyze the functions of *TaGW2* homologues in determining wheat grain weight and protein content traits in hexaploid wheat. These studies all used biolistic bombardment delivery systems. Recently, some groups demonstrated the feasibility of using *Agrobacterium*-delivered Cas9/sgRNA for targeted mutagenesis in the hexaploid wheat. Howells et al. [24] achieved efficient gene targeting of the *TaPDS* gene at rates of 11%–17% for single-genome-targeted guides and 5% for tri-genome-targeted guides. Our research group targeted the *TaDA1* gene and achieved a mutation frequency of 54.17% in T0 transgenic plants using *Agrobacterium tumefaciens*-mediated transformation [25]. Recently, it was reported that the *Agrobacterium*-delivered CRISPR/Cas9 system was used in wheat to target four grain-regulatory genes, with an average editing rate of 10%, and it was confirmed that the mutations were heritable through the T0, T1 and T2 generations [26]. Genome editing has also been successfully applied for the generation of male-sterile wheat lines [27,28]. Okada et al. generated heritable targeted mutations in *Ms1* by using the CRISPR/Cas9 system for the rapid generation of male-sterile hexaploid wheat lines that could be used in hybrid seed production [29].

In recent years, the CRISPR/Cas9 genome editing system has achieved breakthroughs, with an editing efficiency of up to 100% being obtained for rice and maize by a number of laboratories [8,30,31,32]. In contrast, the reported genetic editing efficiency in wheat is much lower than that in rice and maize, reaching a maximum of 54.17%, and only a few genes have been successfully edited. It is worth investigating gene editing in later generations, and transmission patterns need to be studied. In this study, we demonstrated that the CRISPR/Cas9 system could achieve efficient mutagenesis in five target genes of wheat when introduced via *Agrobacterium*-mediated transformation. The *Agrobacterium* binary vector system achieves efficient and heritable targeted mutagenesis in the T1 and T2 generations. The presence of Cas9/sgRNA could cause new mutations in subsequent generations, while mutated transgenic lines without Cas9/sgRNA could retain the mutation type. This provides a new strategy for breeding new wheat cultivars, since it is easy to obtain DNA-free lines by self-crossing the transgenic lines.

## 2. Results

### 2.1. sgRNA Design and Vector Construction

**Figure 1:**
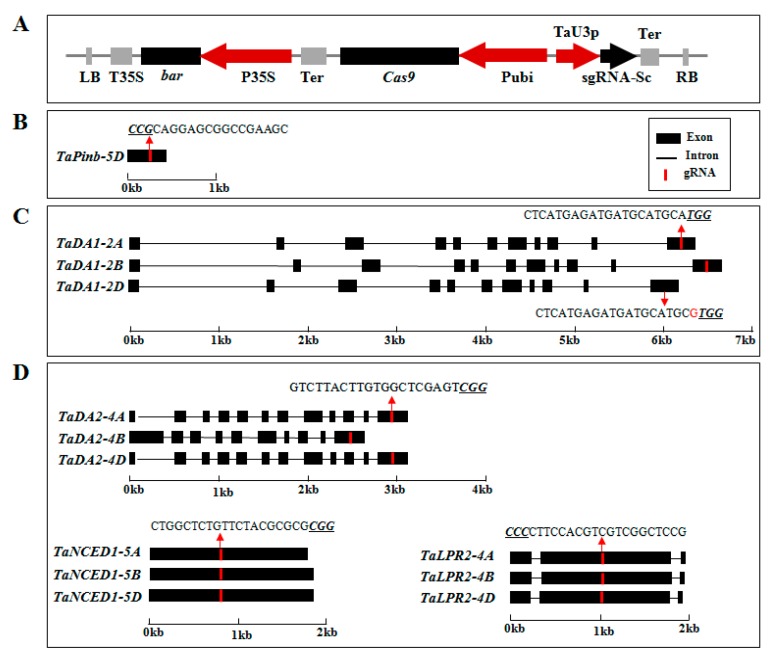
Schematic map of the binary vector and sgRNA selection in the target genes used for wheat transformation. (**A**) The T-DNA region of the binary vector used for genome editing in wheat. Cas9 was expressed with a ubiquitin promoter, and the sgRNA was derived using U3 promoters. (**B**) The gene structure of *TaPinb* and its sgRNA targeting the 5D genome. The *TaPinb* gene is a single-copy gene. (**C**) The gene structure of *TaDA1* and the design of its sgRNA targeting A and B homologues. The sgRNA of the *TaDA1* gene was designed to target the conserved sites of the A and B genomes but showed a mismatch to the D genome at position one at the 3′ end. (**D**) The gene structure of *TaDA2*, *TaNCED1* and *TaLPR2* and the design of their sgRNAs targeting all three homologues. Introns are shown as lines, and exons are shown as black boxes. Target sites are indicated in red. The protospacer adjacent motif (PAM—NGG) sites are underlined and indicated in italics.

Hexaploid wheat presents three sets of subgenomes (AA, BB and DD). Due to the plasticity of these subgenomes, three homologues of some genes are retained, while one or two homologues of other genes are lost. Based on those characteristics, we selected five genes representing singleton, duplex or triplet genes to investigate the editing mode in wheat. Thus, we constructed five independent Cas9-sgRNA vectors that targeted the five wheat genes (i.e., *TaPinb*, *TaDA1*, *TaDA2*, *TaNCED1* and *TaLPR2* genes) (Figure 1A). The *TaPinb* gene is located on chromosome 5DS (Genbank accession number AB262660), and no other copy of this gene was found in the wheat genome. An sgRNA was designed in the coding sequence of the *TaPinb* gene (Figure 1B). The sgRNA targeting the 11th exon of the *TaDA1* gene was designed to target the conserved sites with perfect matches in the A (TraesCSU01G007800) and B (TraesCS2B01G007700) genomes, but there was a mismatch in the D (TraesCS2D01G016900) genome at position one at the 3′ end (Figure 1C). Three sgRNAs targeting the 12th, first and second exons of the *TaDA2* (TraesCS4A01G093500, TraesCS4B01G210900 and TraesCS4D01G211600), *TaNCED1* (Genbank accession number JQ772528) and *TaLPR2* (TraesCS4A01G276100, TraesCS4B01G037600 and TraesCS4D01G035000) genes, respectively, were designed according to the conserved sites of each three homoeologous copies (Figure 1D). Overall, the gene editing experiment involved sgRNAs targeting singleton, duplet and triplet genes (Figure 1, Table S1).

The wheat U3 promoter was selected to drive the sgRNA expression, whereas the expression of Cas9 was driven by the maize ubiquitin promoter (Figure 1A). The functions of the above genes are related to grain quality (*TaPinb*, puroindoline b), grain development (*TaDA1* and *TaDA2*, ubiquitin receptor) and stress-related genes (*TaNCED1*, 9-cis-epoxycarotenoid dioxygenase; and *TaLPR2*, low phosphate response). Knockout mutations of these genes did not cause an easily identifiable phenotype.

### 2.2. CRISPR/Cas9 Induced Highly Efficient Mutagenesis in Four Genes but Not in TaPinb in T0 Plants

After *Agrobacterium*-mediated transformation, the regenerated plantlets were molecularly characterized. A total of 22, 24, 19, 48 and 15 independent T0 transgenic plants were obtained in which *TaPinb*, *TaDA1*, *TaDA2*, *TaNCED1*, and *TaLPR2*, respectively were targeted. The targeted regions were investigated with the corresponding PCR primers and were further confirmed by sequencing (Table S2). The results showed a high mutation efficiency in four target genes. *TaDA1*, *TaDA2*, *TaNCED1*, and *TaLPR2* were all detected with high mutation rates of 54.2% (13 out of 24), 31.2% (6 out of 19), 20.8% (10 of 48) and 46.7% (7 of 15), respectively (Table 1). The highest editing efficiency was observed for the *TaDA1* gene, in which the target region was mutated in 13 lines out of a total of 24 lines. Unfortunately, no mutation was detected in any of the 22 T0 transgenic wheat plants in which *TaPinb* was targeted. The presence of a transgene was confirmed in all 22 lines via the Enviologix QuickStix kit for bar protein. In addition, it has been reported that the GC content of the targeted sites may influence the targeting efficiency. However, the mutation frequency was not in accordance with the GC content of these genes, with the GC content of *TaDA1* being lowest at 43% (Table S1).

### 2.3. Inheritance and Stability of Targeted Mutations Induced by CRISPR/Cas9 in the T1 and T2 Generations

Individual T0 plants from the selected mutant lines were self-pollinated. To investigate whether the targeted mutations that occurred in the T0 plants could be transmitted to the later generations, an analysis was carried out on the progeny of the selected plants. The four selected T0 plants of each target gene used in this study were all positive plants having the Cas9/sgRNA. After the genomic DNA was extracted, PCR amplification and Hi-TOM sequencing were performed for mutation analysis. For the *TaPinb* gene, we did not detect CRISPR/Cas9-induced mutations at the sgRNA target site in the T0 plants. To further investigate the possible reason for the unsuccessful mutation targeting, we randomly chose six positive T1 plants from each independent transgenic event for mutation detection. The results showed that some T1 plants had detectable mutations. Ten plants from a total of 132 plants from nine independent lines exhibited mutations. The editing efficiency was 7.6% (Table 2).

For the other four genes, approximately 20 seeds from each of the four selected mutated T0 plants were germinated and grown in the greenhouse. For the T1 plants, we wanted to find the inheritance patterns of the mutations in both positive and negative plants. Some T1 lines that either exhibited a T-DNA insertion or not by bar detection were both selected for analysis. Genotype analysis showed that heritable targeted mutations were present in the T1 progeny. The total mutation rates of *TaDA1*, *TaDA2*, *TaNCED1* and *TaLPR2* were 97.6%, 74.3%, 75% and 86.9%, respectively. For each T1 line, the editing efficiency of the four genes ranged from 61.5% to 100%. The editing efficiency in four T1 lines reached 100% for *TaDA1*-15, *TaDA1*-17, *TaLPR2*-1 and *TaLPR2*-17 reached 100% (Table 2). The proportion of the T1 generation showing 100% editing efficiency for the four genes was 31.3%.

Five to ten T2 transgenic lines from the four mutant lines that carried three genotypes (homozygous, heterozygous and chimaeras) either exhibited a T-DNA insertion or not were selected for analysis. Approximately 11–20 seeds of each line were germinated for DNA extraction and sequencing. It is worth noting that the editing efficiencies of the selected T2 transgenic lines were all significantly increased. The proportion of transgenic lines with 100% editing efficiency increased to 66.9%, which was significantly higher than that in the T1 generation according to Fisher’s exact test, with a *p*-value = 1.06 × 10^−8^. Among all 31 selected T2 transgenic lines, 21 lines exhibited 100% mutation rates. *TaDA1*-edited and *TaLPR2*-edited plants of the T2 generation were both detected in six lines among the selected seven lines exhibiting 100% editing efficiency. The total mutation rates of *TaDA1*, *TaDA2*, *TaNCED1* and *TaLPR2* were 99.2%, 79.1%, 84.1% and 96.1% respectively (Table 2), which were higher than the rates in the T1 generation.

The editing bias of each subgenome was further investigated. Some target genes showed a mutation preference in the subgenomes across generations. For example, *TaLPR2* exhibited a smaller proportion of B genome editing compared with the other genomes (A and B) in T0, T1 and T2 generations, and *TaDA2* presented higher editing rates in the D genome in the T1 and T2 generations (Figure S1).

These results showed that the targeted mutagenesis induced by CRISPR/Cas9 is stably inherited in the progeny of hexaploid wheat.

### 2.4. CRISPR/Cas9-Induced Mutation Types Across Generations

After *Agrobacterium*-mediated stable transformation, independent T0 transgenic plants were obtained, and target mutations were analyzed in the T0, T1 and T2 lines. A number of T1 plants derived from the same T0 plants were investigated in the mutation analysis (Table 2 and Table S3). Some of the T2 plants generated from the corresponding T1 lines were selected on the basis of whether they presented genotypes (homozygous, heterozygous and chimaeric) with or without the transgene (Table 2 and Table S4). Most of the gene mutations were stably passed on to the next generation, though some mutations occurring in T0 were lost, and some new mutations were generated in the progeny. We detected the T-DNA region by bar detection. When the Cas9 protein was removed, for example, the 14–6 and 15–8 of *TaDA1*, 3–12 and 60–3 of *TaDA2*, 98–3 of *TaNCED1*, and 54–23 of *TaLPR2* (Table S4), of these lines had the same genotype as the T1, or different genotypes from T1 because of segregation in T2. The editing types of these lines were also stably inherited from the T1, except for a few lost types (Table 2). All of the insertion mutations occurred at the 4th base upstream of the PAM (protospacer adjacent motif) site, and nearly all of the 1 bp deletions were also located at this position. Relatively, longer deletions also occurred, including deletions at this site.

**Figure 2:**
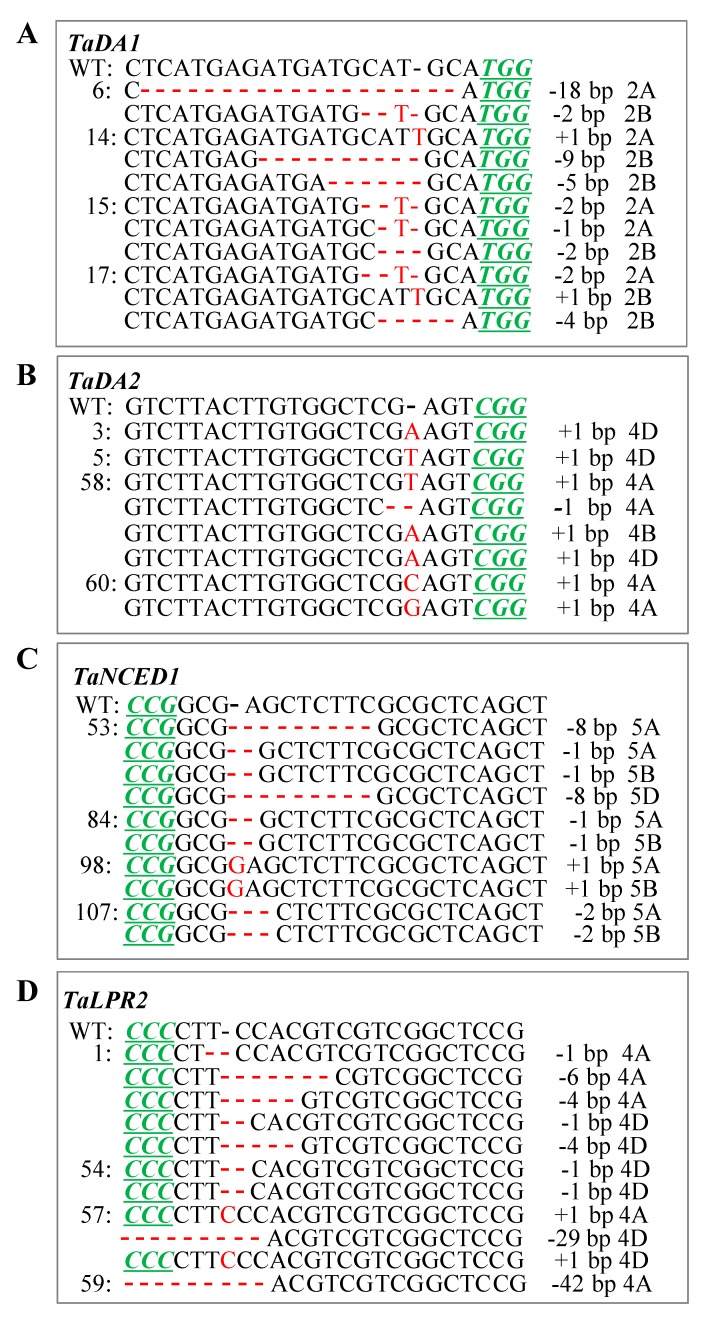
CRISPR/Cas9-induced targeted mutagenesis of the target genes *TaDA1* (**A**), *TaDA2* (**B**), *TaNCED1* (**C**) and *TaLPR2* (**D**) in the T0 plants. The mutation sites are indicated in red. The PAM (NGG) sites are underlined and indicated in italics in green.

**Figure 3:**
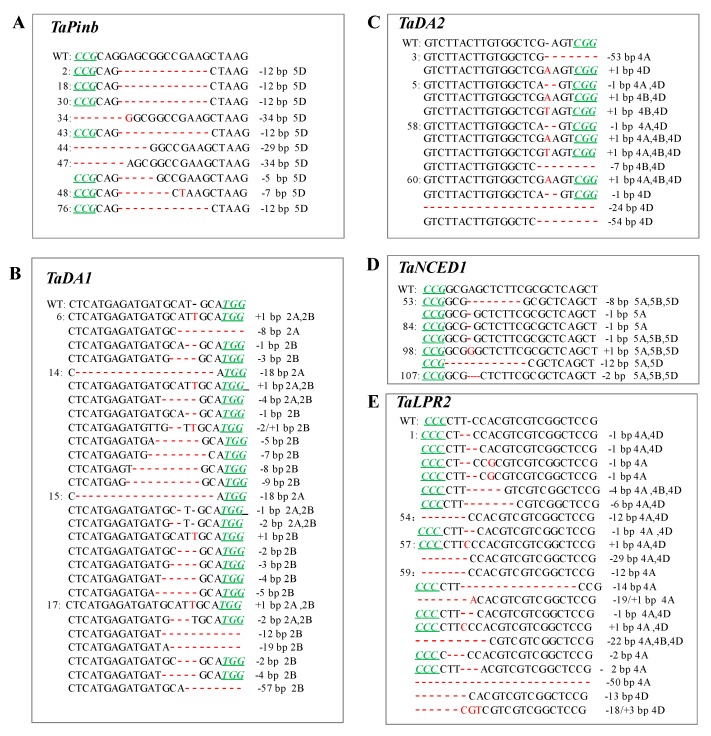
CRISPR/Cas9-induced targeted mutagenesis of the target genes *TaPinb* (**A**), *TaDA1* (**B**), *TaDA2* (**C**), *TaNCED1* (**D**) and *TaLPR2* (**E**) in T1 plants. The mutation sites are indicated in red. The PAM (NGG) sites are underlined and indicated in italics in green.

**Figure 4:**
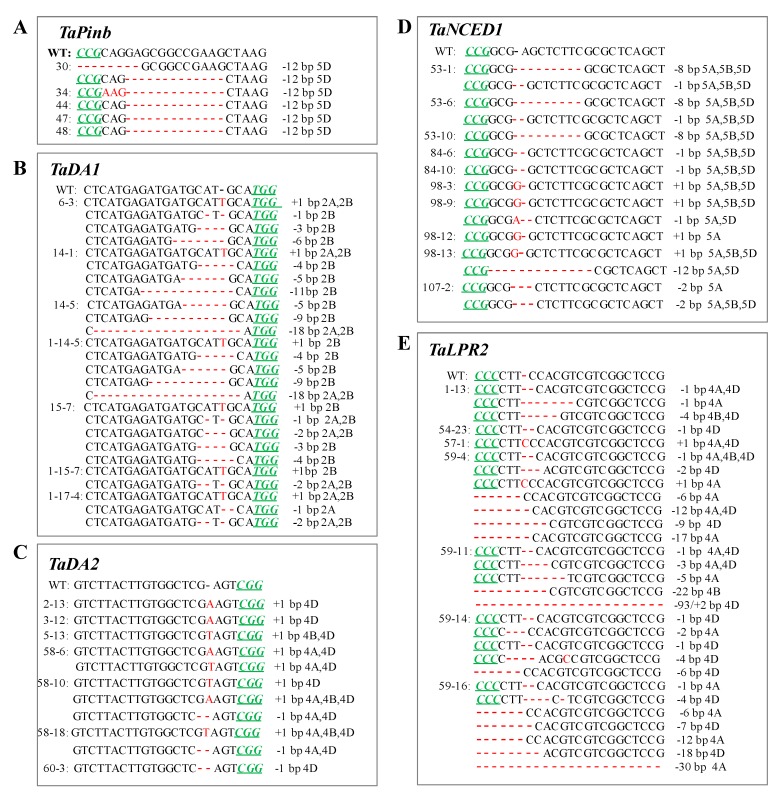
CRISPR/Cas9-induced targeted mutagenesis of the target genes *TaPinb* (**A**), *TaDA1* (**B**), *TaDA2* (**C**), *TaNCED1* (**D**) and *TaLPR2* (**E**) in T2 plants. The mutation sites are indicated by red. The PAM (NGG) sites are underlined and indicated in italics in green.

For the *TaPinb* gene, the observed mutation types included only deletions, such as 5d, 7d, 12d, 29d and 34d in T1 (Figure 3A, Table 2), but only 12d in T2 (Figure 4A, Table 2). For the *TaDA1* gene, the mutation types included both insertions, such as 1i in T0, T1 and T2 generations, and deletions, such as 1d, 2d, 4d, 5d, 9d and 18d in T0 (Figure 2A, Table 1); 1–5d, 7–9d, 12d, 18d, 19d and 57d in T1 (Figure 3B, Table 2); and 1–6d, 9d, 11d and 18d in T2 (Figure 4B, Table 2). For the *TaDA2* gene, only 1i and 1d were detected in T0 (Figure 2B, Table 1), while more variations were detected in T1, including 1i and 1d, 7d, 24d, 53d and 54d (Figure 3C, Table 2). Because T2 plants were partially selected from the T1 lines, only 1i and 1d were detected in T2 plants (Figure 4C, Table 2). For *TaNCED1*, the variation types were stable and included 1i, 1d, 2d and 8d in T0 plants (Figure 2C, Table 1), while 12d was additionally found in T1 plants (Figure 3D, Table 2), and the same variations were found in T2 (Figure 4D, Table 2). For the *TaLPR2* gene, the mutational variations were complex. A 1 bp insertion was detected in T0, T1 and T2 generations (Figure 2D, Figure 3E and Figure 4E, Table 1 and Table 2). Regarding deletions, 1d, 4d, 6d, 29d and 42d were detected in T0 (Figure 2D). In addition, 12–14d, 18d, 19d, and 50d appeared in T1 (Figure 3E, Table 2), and novel mutations, such as 3d, 5d, 7d, 9d, 17d, 22d, 30d and 93d occurred in T2 (Figure 4E, Table 2). There were differences in the mutation types in each subgenome for *TaDA1*, *TaDA2* and *TaLPR2*, while *TaNCED1* exhibited similar mutation types among the three subgenomes (Figure S2).

**Figure 5:**
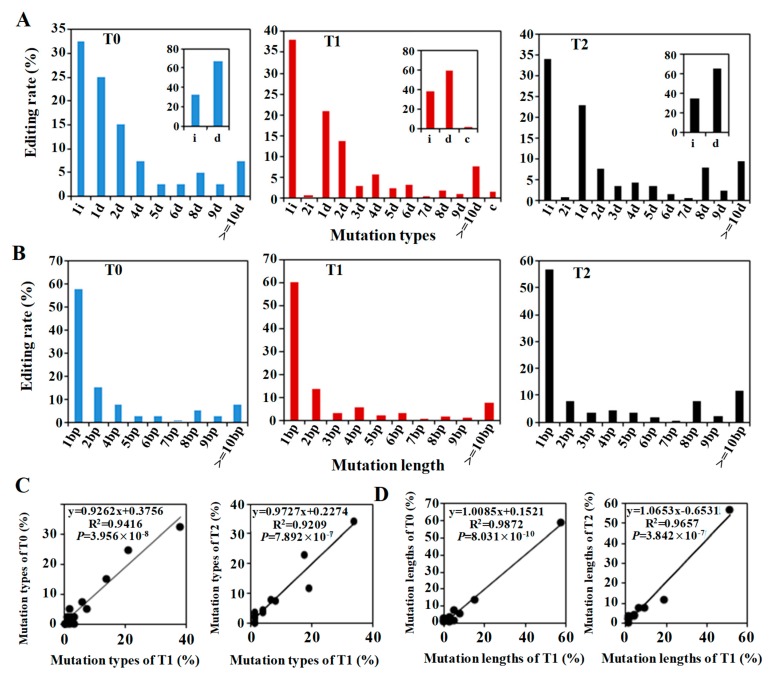
The distributions and correlations of the mutations across generations. (**A**) Distribution of mutation types in the T0, T1 and T2 generations. (**B**) Distribution of the mutation lengths in T0, T1 and T2 generations. (**C**) Correlation of mutation type frequency between the T0 and T1 generations. (**D**) Correlation of mutation type frequency between the T1 and T2 generations. The correlation was analyzed by Pearson’s method. i, insertion; d, deletion; c, combined mutation types.

There was a very similar trend in the mutation types between the T0, T1 and T2 generations (Figure 5A). The types of mutations varied among the target regions. Among the five different target genes, the mutation types were similar. Two mutation types, insertions and deletions, were present in all the gene targeting events, especially 1 bp insertions and 1 bp deletions. The other mutation types included a series of short deletions, such as 2d, 4d and 5d. Most of the deletions were short, but there were also longer deletions that ranged from ten to ninety-three base pairs (Figure 2, Figure 3 and Figure 4). Among the wide variety of mutation types, 1 bp insertions presented the highest proportion. However, the proportion of total deletions was higher than that of total insertions when all the mutation events were summed. It is worth noting that the mutation types found in the T1 and T2 generations were both increased in number and were more abundant in the T0 lines with the presence of the transgene, such as the 2 bp insertions and several different lengths of deletions. In addition to the majority of the mutation types in T0 plants, a variety of new mutations also emerged. Some mutations occurring in T0 were lost in the subsequent generations. In the absence of the transgene in some T2 plants, the targeted mutations were relatively stable and regular. The mutation genotypes of some T1 lines were not changed and were stably passed to the T2 generation without the CRISPR/Cas9 system, such as the *TaDA2*-3-2, *TaDA2*-60-3 and *TaNCED1*-98-3 (Table 2). The mutation types of the T0 and T1 plants presented a very high-frequency correlation by the Pearson method, with R^2^ = 0.9416 (*p*-value = 3.956 × 10^−10^), as did the mutation types of the T1 and the selected T2 plants, with R^2^ = 0.9657 (*p*-value = 3.842 × 10^−7^) (Figure 5C).

The distribution of mutation lengths was similar to that of mutation types in the T0, T1 and T2 lines (Figure 5B). The mutation length of 1 bp, including both 1 bp insertions and 1 bp deletions, was the most common, followed by the 2 bp mutation length, including of 2 bp insertions and 2 bp deletions. The distribution of other different deletion lengths was the same as that of the mutation types. Similarly, some mutation length types were lost, and new mutation length types arose across the generations. Mutation length in the T0 and T1 plants exhibited a very high-frequency correlation according to the Pearson method, with R^2^ = 0.9872 (*p*-value = 8.031 × 10^−8^), as did the mutation length of the T1 and the selected T2 plants with R^2^ = 0.9209 (*p*-value = 7.892 × 10^−^^7^) (Figure 5C).

### 2.5. Editing Frequency of the Mutant Genotypes across the Generations

**Figure 6:**
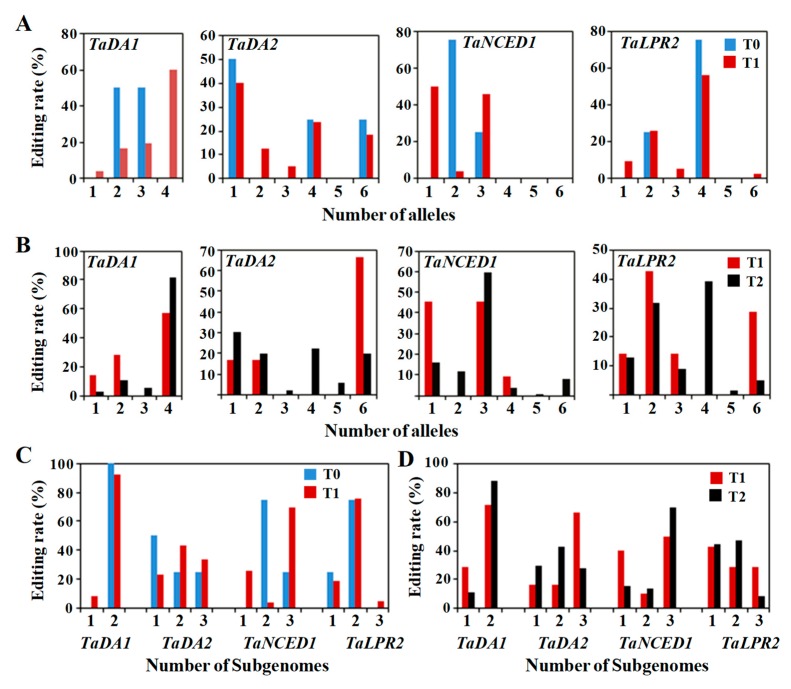
The editing rates of alleles and subgenome numbers of the target genes in each generation. (**A**) Editing frequency of the alleles in T0 and T1 generations. (**B**) Editing frequency of alleles in the four selected T1 plants and their T2 progeny. (**C**) Editing frequency of the subgenomes in the T0 and T1 generations. (**D**) Editing frequency of subgenomes in the T0 and T1 generations in the four selected T1 plants and their T2 progeny.

Hexaploid wheat generally presents three homoeologous subgenomes (A, B and D) and includes six alleles. In this study, we used a singleton of *TaPinb*, a duplet of *TaDA1* and three triplets of *TaDA2*, *TaNCED1* and *TaLPR2* for targeted mutagenesis in wheat. Among these genes, the most distinctive situation was found for the *TaDA1* gene, which was found in all three subgenomes, although the sgRNA was designed to target the A and the B but not the D genome. The genotypes of the mutant plants were analyzed. The editing efficiency of two groups of target genes, duplets and triplets, is shown in Figure 6. In the presence of the transgene, a number of new mutations occurred in the T1 and T2 generations compared to the T0 generation. The proportion of editing in the targeted subgenomes and alleles continued to increase in the progenies. Homozygous mutants could be obtained whether the target gene was present in a single copy, two copies or three copies in the hexaploid wheat genome. For example, the *TaPinb* lines with a singleton genome were first found to exhibit heterologous mutations in the T1 generation (Table S3) and to be homozygous in the T2 generation (Table S4).

In the four selected independent *TaDA1* T0 lines 6, 14, 15 and 17, the two targeted subgenomes presented mutations in the A and B subgenomes, but only two or three alleles with homologous mutations were found for one subgenome (Figure 6A,C). As expected, no mutations were detected in the D genome because of the 1 bp mismatch in the sgRNA region. In the progeny of T1 and T2, mutations were detected in one or two subgenomes, and included one, two, three or four alleles (Figure 6B,D). These mutant T0 lines were determined to be homozygous and to show a 60.2% editing rates in the T1 generation. Homozygous mutations occurred in the T1 lines, such as *TaDA1*-15 and *TaDA1*-17, with the genotype of HomAHomB (Table S3). The editing rate of T2 homozygotes increased to 81.3%, which was higher than that of T1 plants.

The results of using a tri-genome targeting sgRNA for the *TaDA2, TaNCED1* and *TaLPR2* genes showed some differences in the simultaneous editing of three homoalleles in hexaploid wheat. In the T0 generation, the *TaDA2* and *TaNCED1* lines exhibited mutations in three subgenomes, but all six alleles of the three subgenomes were edited only for the *TaDA2* gene, as was found for *TaDA2*-58, whose genotype was BiaAHomBHomD. Meanwhile, the *TaNCED1* mutants were heterozygous for all three homoalleles (Figure 6A,C, Table S3). New editing types also occurred in other subgenomes because of the presence of the transgene (T-DNA). In the presence of Cas9, the four genes that were mutated in the T0 plants were successfully edited in all subgenomes which were edited in the T1 generation (Table S3). The number of homozygotes and chimaeras continued to increase in the T2 generation (Table S4). Similar to that which occurred for the *TaLPR2* genes, the T0 plants showed mutations in only one or two subgenome alleles, but exhibited tri-genome-mutated alleles in T1 lines, such as *TaLPR2*-1 and *TaLPR2*-59 (Figure 6C). The *TaLPR2* gene was homozygous in the T1 generation (Figure 6A). *TaNCED1*-84, *TaNCED1*-98 and *TaNCED1*-107 were edited in the A and B genomes of the T0 plants, and all tri-genome alleles presented mutations in the T1 plants (Figure 6A,C, Table S3). However, homozygous *TaNCED1* mutants for all three homoalleles were detected in the T2 generation (Figure 6B,D, Table S4).

These results suggest that a number of the T0 plants exhibited mutations in a single genome (A, B or D) or two subgenomes (A, B; A, D; or B, D), but novel mutations were generated in their subsequent generations due to the continuous activity of the Cas9/sgRNA complex. We were able to obtain homozygous mutations in hexaploid wheat in the T0 generation or its T1 and T2 progeny.

### 2.6. Off-Target Analysis of Targeting sgRNAs

To further assess the off-target effects of the CRISPR/Cas9 system, the target sequence plus the PAM motifs of each gene were used to search the wheat reference genome. Six potential off-target sites with 2 bp or 3 bp mismatches compared to the target sequence were selected for off-target analysis. Twenty-four T2 progeny plants were used for each potential target site for mutation detection. DNA was extracted, and PCR amplification of putative off-target sites was performed. The potential off-target effects and the PCR primers used for amplifying the potential off-target sites are listed in the supplementary material (Table S5). The sequencing results showed that no mutations were observed at those sites. Our results showed that the CRISPR/Cas9 system was highly specific for gene editing in transgenic wheat plants.

## 3. Discussion

**Figure 7:**
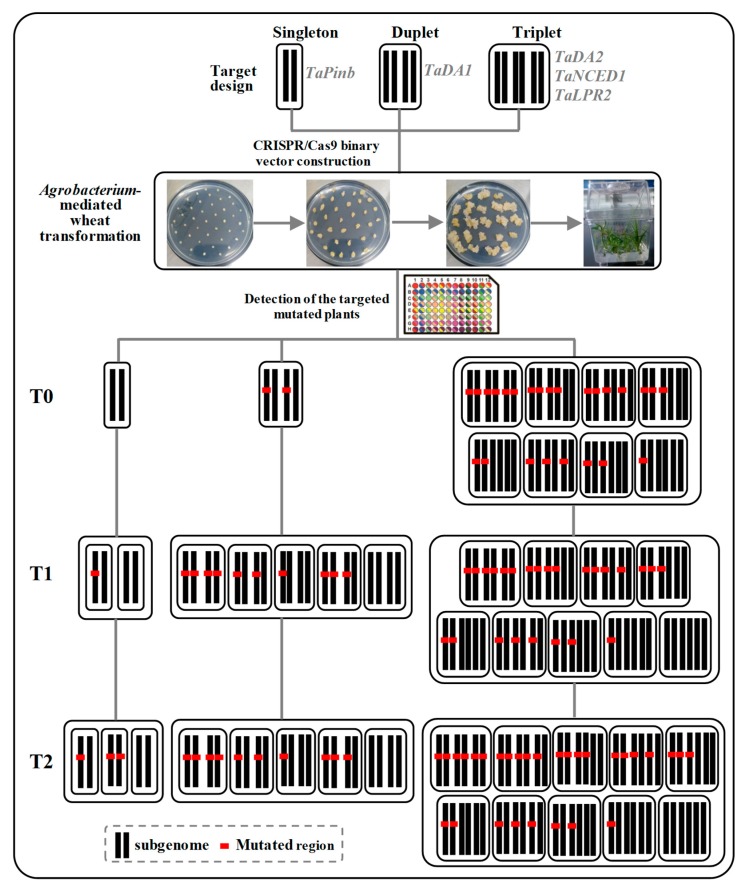
Schematic overview of the procedure for the generation of mutations and mutation detection in T0, T1 and T2 wheat plants. We targeted five genes in wheat using the CIRSPR/Cas9 system with *Agrobacterium-mediated* delivery. The transgenic wheat plants were then evaluated with the Hi-TOM mutation detection kit. A high mutation efficiency could be obtained in T0 plants, and the mutations could be stably transmitted to the T1 and T2 progenies.

Compared to *Arabidopsis*, rice and maize, all of which are diploids, common wheat (*Triticum aestivum* L.) exhibits a complex genome structure involving a combination of three different genomes (AA, BB and DD), which makes it an important model for studying and optimizing genome editing systems in polyploid plants. Here, we targeted five genes in wheat using the CIRSPR/Cas9 system. A schematic overview of the experimental workflow is provided in Figure 7, which includes the steps from the sgRNA design to plant mutation detection across generations. We reported that the CRISPR/Cas9 system can efficiently achieve specific targeted mutagenesis with an editing efficiency of 20.8%–54.2% in T0 plants when delivered by *Agrobacterium*-mediated wheat transformation. The mutation types were heritable and novel mutations could be produced in the T1 and T2 generations without detecting off-target mutations.

Biolistic bombardment and the *Agrobacterium*-mediated transformation are the two major approaches for wheat transformation. In addition to the designing of the CRISPR/Cas9 system, including the promoters used and the selection of sgRNA, the editing efficiency also depends on the delivery of that complex. Most of the previous genome editing experiments related to using the CRISPR/Cas9 system in wheat were conducted by biolistic transformation [13,21,33,34]. The main problem is that the biolistic transformation requires hundreds of wheat embryos, and for most of the editing events that occur during transient expression of the CRISPR/Cas9 system, the CRISPR/Cas9 and selection marker cassette are not integrated into the genome. This situation could result in many transgene copies, which are associated with a high frequency of gene silencing, and this may be related to the low editing rate observed. It was very hard to detect mutagenesis, which is a labor intensive, time consuming and financially costly process. Compared to this method, *Agrobacterium*-mediated transformation with the CRISPR/Cas9 system is more repeatable and less costly in terms of both money and labor. Additionally, since the T-DNA is integrated into the genome, mutations were induced with high efficiency and show stable heritability in wheat. We had tested the *Agrobacterium*-mediated transformation with a series of different combinations of endonucleases (zCas9, pCas9, AteCas9, proCas9 and hCas9) and sgRNA promoters (OsU3, OsU6, TaU3 and TaU6). The results showed that the use of OsU3, OsU6 and TaU6 with their binary vectors does not work in the wheat plants. Only the combination of the TaU3 promoter and the zCas9 has the highest editing efficiency, which we used in this study.

The allohexaploid genome of wheat exhibits many genes with three copies on homoeologous chromosomes. This genetic redundancy impedes genetic functional studies. The phenotypical effects of mutations within a single homologue are frequently masked by other gene copies. We need to investigate the contribution of mutations in each homoeologous gene and their combined contributions to phenotypic variation in polyploid wheat. In this study, we designed three types of editing strategies for five wheat genes, including the use of an sgRNA targeting the 5DS genome of the *TaPinb* gene; a single sgRNA targeting both the 2A and 2B genomes for the *TaDA1* gene; and three sgRNAs targeting the three subgenomes for the *TaDA2*, *TaNCED1* and *TaLPR2* genes. Our results confirmed a high mutation efficiency in the T0 generation. However, not all target sites were mutated in T0 plants. The similar editing case of the *TaPinb* lines, where mutation could be detected in T1 and T2 but not in T0, have been reported in previous studies [35,36]. The mutations that were not detected in T0 could be due to the limited detection of rare mutation events in chimeric leaf tissues. The mutagenesis efficiency was variable, due to the sgRNA characteristics and CRISPR/Cas9 being continuously present across number of generations [36]. In our present study, the mutation rates in the targeted genes (subgenomes and alleles) continued increasing in the progenies. We were able to obtain homozygous mutations in hexaploid wheat in the T0 generation or its T1 and T2 progeny.

The editing types and the inheritance of targeted mutagenesis were similar to those in rice [37,38], which was not related to the sgRNA-targeted subgenome number. However, some genes exhibited mutation preference in the subgenomes across generations. The mutation types in each subgenome were also different except for *TaNCED1.* The targeted mutations could be stably inherited in the next generation, and the editing efficiency of each mutant line increased significantly across the generations. The mutation types occurring in the plants may be lost in the next generation, which may result from somatic mutations being lost in the progeny. In addition, it has been reported that different mutations can be detected in samples of different tissues [39]. Mutations in tissues that we did not examine may have been overlooked because we examined only the target sequence in a single leaf sample. In addition, the PCR amplification efficiency has important effects. New mutations could be generated in progeny. The existence of Cas9/sgRNA continued to play roles in editing the target sequences in the T1 and T2 generations, with an increasing mutational efficiency. Therefore, increases and decreases in the present mutation types were frequently found in the progeny of T1 and T2.

The existence of chimaeras is an important issue for the CRISPR/Cas9 system. Our results showed that chimaerism did occur, and the WT target sequences with an abundance of heterozygotes and chimaeras could continue to mutate in the T1 and T2 generations. Genome editing could occur at any time and in any cells during the plant life cycle, giving rise to the question of whether the mutations could be present in germ cells and transmitted to the offspring. A previous study revealed that the adoption of a gametophyte-specific promoter to control the expression pattern of Cas9 was an effective way to solve the chimeric mutant problem [39].

It seems that not all sgRNAs are highly active in targeted mutagenesis in plants. The mutation types and mutation rates seemed to be different across the different target genes. One of the possible reasons for this may be that the activity of the CRISPR/Cas9 system in different transgenic events depends on where the CRISPR/Cas9 transgene is inserted into the plant genome [38]. The position and the base composition of the selected sgRNA may also impact the function of the CRISPR/Cas9 system. Additionally, it has been reported that chromatin accessibility is associated with CRISPR-Cas9 efficiency in the human cells and zebrafish [40,41,42]. The editing efficiency may be related to the chromatin state of the target genes.

In this study, the mutated plants did not show an obvious phenotype. That may be related to the existence of heterozygotes and chimaeras, and gene redundancy in the polyploid wheat. The complex genome composition of wheat makes it difficult to edit all alleles simultaneously. In addition, the selected five genes, except *TaPinb* were all newly characterized genes from wheat; their functions were not identified in wheat. The functions of the genes are related to grain quality (*TaPinb*, puroindoline b), grain development (*TaDA1* and *TaDA2*, ubiquitin receptor) and stress-related genes (*TaNCED1*, 9-cis-epoxycarotenoid dioxygenase; and *TaLPR2*, low phosphate response). Knockout mutations of these genes did not cause an easily identifiable phenotype. Thus, more experiments are needed, such as grain quality and abiotic stresses treatment to characterize the gene function.

In the present study, the combination of the *Agrobacterium*-mediated transformation method with the CRISPR/Cas9 system significantly improved mutagenesis efficiency significantly; and the integrated CRISPR/Cas9 cassette could be transferred to other cultivars via conventional breeding, and additional mutagenesis would occur in the new cultivars. In our study, the CRISPR/Cas9 transgene still exhibited high editing activity in the T1 and T2 generations. Only one transformation process was necessary and could result in mutants in different cultivars. Moreover, because the editing sites and the insertion of the CRISPR/Cas9 cassette occur at different loci, we do not worry about the dangers of DNA (CRISPR/Cas9 cassette)-harboring editing lines. It is easy to obtain DNA-free lines by self-crossing the transgenic lines. Therefore, this approach provides a new strategy for the breeding and promotion of new wheat cultivars.

## 4. Material and Methods

### 4.1. sgRNA Design and Plasmid Construction

The target sequences were designed and selected using CRISPRdirect (http://crispr.dbcls.jp/) and CRISPOR (http://crispor.tefor.net/) to minimize off-target effects. The oligonucleotide primers used are listed in Table S2. The recombinant plasmid was generated by introducing the guide RNA into the binary vector pBUE411, which is modified with the wheat TaU3 promoter and guides the RNA scaffold (Table S1). The Cas9-sgRNA expression vectors were constructed as previously described [43].

### 4.2. Plant Materials and Genetic Transformation

The constructed vector was introduced into the *Agrobacterium tumefaciens* strain EHA105. The wheat variety Fielder was used. Immature wheat embryos were isolated and *Agrobacterium*-mediated transformation was performed according to Zhang et al. [25]. The plants were grown in greenhouses at a temperature of 20 °C with a light intensity stronger than 60,000 lx and a night temperature of 16 °C. Transgenic lines were selected based on BASTA resistance. Transgenic wheat plants were identified with the Enviologix QuickStix kit for Bar protein (ENVIROLOGIX, Portland, ME, USA). The T1 and T2 generation progeny were collected from independent T0 lines.

### 4.3. Detection of the Targeted Genome Editing Events

Genomic DNA from the leaves of transgenic wheat plants was extracted using the Tiangen DNAquick Plant System (Tiangen, Beijing, China). NGS library construction and next-generation sequencing were performed using the Hi-TOM Gene Editing Detection Kit (Novogene, Beijing, China) [44]. Specific PCR primers were designed and used for PCR amplification of relevant regions flanking the target sites (Table S2). Then barcoding PCR was carried out for library construction. Equal amounts of PCR products were mixed in a pool. These samples were used for Illumina sequencing at the Novogene Company (Beijing, China). Amplified DNA fragments of target genes were sequenced and aligned with the BioEdit program, which was used to analyse the nucleotide sequencing results. Indels (insertions and deletions) occurring at the targeted sites of the target genes were considered mutations.

### 4.4. Off-Target Analysis

The potential off-target effects of CRISPR/Cas9 in common wheat were identified using the BLASTN tool against the wheat genome sequence (URGI: https://urgi.versailles.inra.fr/blast/?dbgroup=wheat_all&program=blastn) by searching for the seed sequence plus PAM. Hits with fewer than three mismatches were chosen for amplification and analysis by using specific primers (listed in Table S5). Potential off-target sites were amplified and sequenced using the Hi-TOM Gene Editing Detection Kit (Novogene, Beijing, China).

## Figures and Tables

**Table 1:** Targeted mutagenesis in T0 plants.

Target Gene	Number of Plants Examined	Mutation Line	Mutation Rate (%)	Mutation Types (bp)
*TaPinb*	22	0	0	-
*TaDA1*	24	13	54.2	1i, 1d, 2d, 4d, 5d, 19d
*TaDA2*	19	6	31.2	1i, 1d
*TaNCED1*	48	10	20.8	1i, 1d, 2d, 8d
*TaLPR2*	15	7	46.7	1i, 1d, 3d, 4d, 6d, 29d, 42d

i, insertion; d, deletion; -, No mutation.

**Table 2:** Mutation rates and mutation types in the T1 and T2 generation.

Target gene	T1 Line	Number of Plants Examined	Number of Mutated Plants	Mutation Rates (%)	Mutation Types (bp)	T2 Line from T1	Number of Plants Examined	Number of Mutated Plants	Mutation Rates (%)	Mutation Type (bp)
*TaPinb*	1-22	132	10	7.6%	5d, 7d, 12d, 29d, 34d	47	21	1	4.76	12d
						30	21	15	71.4	12d
						34	21	2	9.5	12d
						44	21	2	9.5	12d
						48	12	1	8.3	12d
*TaDA1*	6	13	12	92.3	1d, 3d, 1i	6-3	17	17	100	1i, 1d, 3d, 6d
	14	21	20	95.2	4d, 5d,7d, 9d, 18d, 1i	14-1	19	19	100	1i, 4d, 5d, 11d,
						14-15	19	19	100	5d, 9d, 18d
						14-6	19	18	94.7	1i, 4d, 5d, 9d, 18d
	15	25	25	100	1d, 2d, 3d, 4d, 1i	15-7	15	15	100	1i, 1d, 2d, 3d, 4d
						15-8	15	15	100	1i, 2d
	17	26	26	100	1d, 2d, 4d, 12d, 19d, 57d, 1i	17-4	16	16	100	1i, 1d, 2d
*TaDA2*	3	19	15	78.9	53d, 1i	3-12	17	13	76.5	1i
						3-13	13	12	92.3	1i
	5	13	8	61.5	1d, 1i	5-13	22	16	72.7	1i
	58	20	18	90	1d, 7d, 1i	58-6	21	21	100	1i, 1d
						58-10	16	16	100	1i, 1d
						58-18	12	12	100	1i, 1d
	60	22	14	63.6	1d, 24d, 54d, 1i	60-3	14	1	7.1	1d
*TaNCED1*	53	11	7	63.6	1d, 8d	53-1	18	16	88.9	1d, 8d
						53-6	20	20	100	1d, 8d
						53-10	15	15	100	8d
	84	20	14	70	1d	84-6	20	10	50.0	1d
						84-10	14	14	100	1d
	98	17	16	94.1	1i, 12d	98-3	8	8	100	1i
						98-9	16	16	100	1i, 1d
						98-12	18	14	77.8	1i
						98-13	11	11	100	1i, 12d
	107	16	11	68.8	2d	107-2	11	3	27.3	2d
*TaLPR2*	1	22	22	100	1d, 4d, 6d, 2i	1-13	20	20	100	1d, 4d, 6d
	54	23	23	100	1d, 12d	54-23	20	20	100	1d
	57	18	14	77.8	29d, 1i	57-1	18	13	72.2	1i
	59	21	14	66.7	1d, 2d, 6d, 12d, 13d, 15d, 18d, 22d, 30d, 50d, 53d, 1i	59-4	16	16	100	1d, 1i, 2d, 6d, 9d, 12d, 17d
						59-11	20	20	100	1d, 3d, 5d, 22d, 93d2i
						59-14	15	15	100	1d, 2d, 4d, 6d
						59-16	18	18	100	1d, 4d, 6d, 7d, 12d, 18d, 30d

i, insertion; d, deletion.

## Supplementary Materials

Supplementary materials can be found at https://www.mdpi.com/1422-0067/20/17/4257/s1.

## Abbreviations

CRISPR	Clustered Regularly Interspaced Short Palindromic Repeats
DSB	Double-Stranded Breaks
NHEJ	Non-Homologous End Joining
HDR	Homology-Directed Repair
PAM	Protospacer Adjacent Motif
TALEN	Transcription Activator-like Effector Nuclease
ZFN	Zinc Finger Nucleases
