# Supplementary material for: Highly Efficient and Heritable Targeted Mutagenesis in Wheat via the Agrobacterium tumefaciens-Mediated CRISPR/Cas9 System
Source: Int J Mol Sci. 2019 Aug 30;20(17):4257. doi: 10.3390/ijms20174257 (PMC6747105; doi:10.3390/ijms20174257)
Supplement: Supplementary file 1 [file ijms-20-04257-s001.pdf]

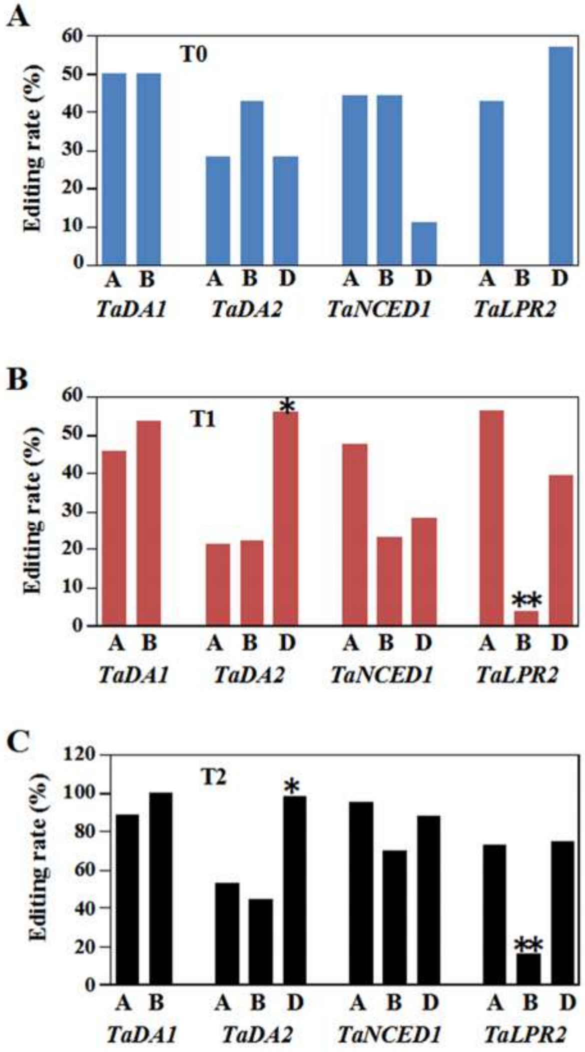

Figure S1. Editing rates of the target genes in each subgenome across the T0 (**A**), T1 (**B**) and T2 (**C**) generations. Asterisks indicate a significant difference from the other subgenomes of each target gene at the *, P-value < 0.05 level of significance or **, the P-value < 0.01 level of significance by Fisher's exact test.

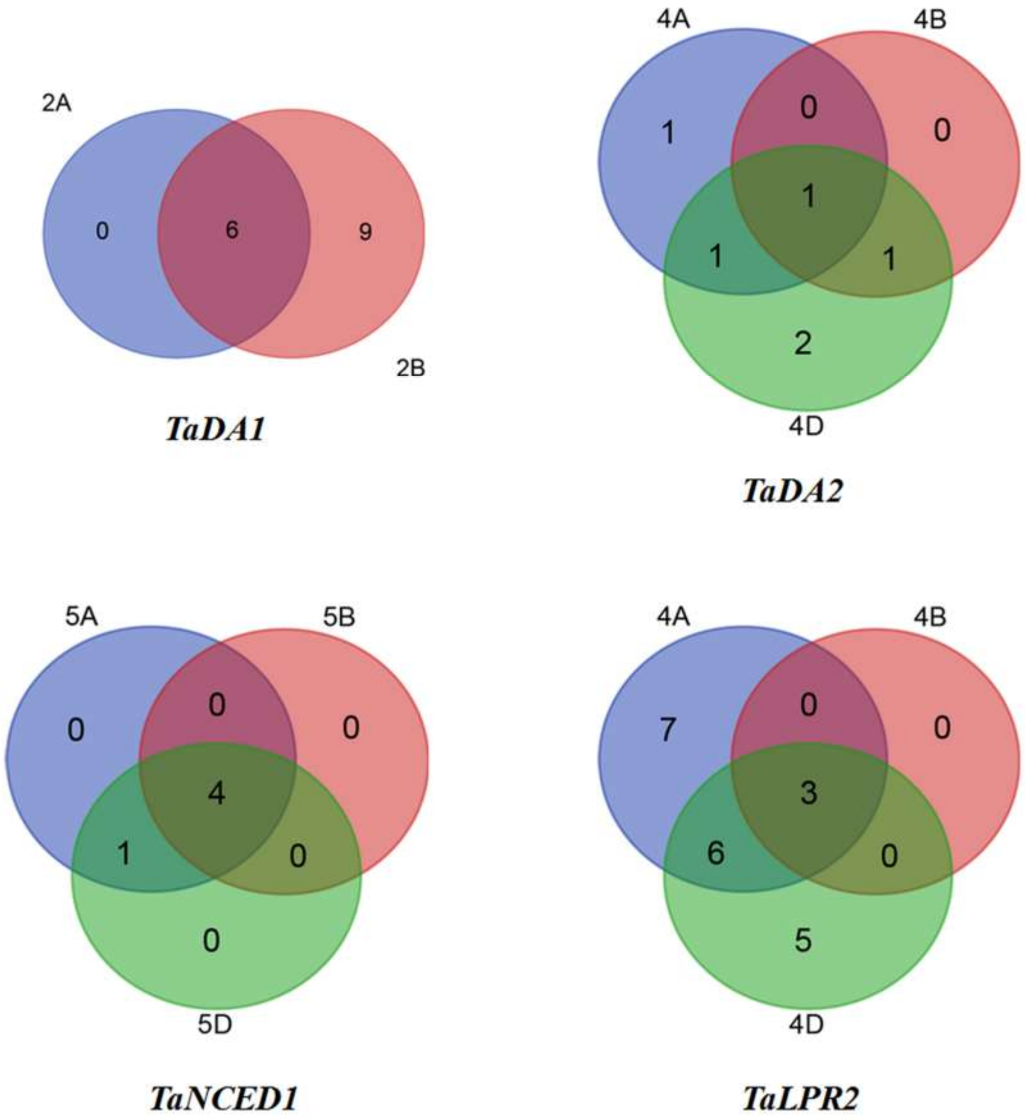

Figure S2. Overlap of editing types in each subgenome among the target genes.

Table S1. sgRNA selection.

Target Gene	sgRNA Sequence (5′-3′)	GC Content (%)	Targeted Genome
TaPinb	CTTAGCTTCGGCCGCTCCTG	65%	5D
TaDA1	ACTCATGAGATGATGCATGCA	43%	2A, 2B
TaDA2	GTCTTACTTGTGGCTCGAGT	50%	4A, 4B, 4D
TaNCED1	GCTGAGCGCGAAGAGCTCGC	70%	5A, 5B, 5D
TaLPR2	CGGAGCCGACGACGTGGAAG	70%	4A, 4B, 4D

Table S2. Primers for mutation detection in transgenic wheat plants.

Target Gene	Oligo Sequence (5′-3′)
pinb-F	ggagtgagtacggtgtgcTGTAGCGAGCACAACCTT
pinb-R	gagttggatgctggatggGTGGTGCTATCTGGCTCA
DA1-F	ggagtgagtacggtgtgcGACTTTGCGAGTCTCTAAT
DA1-F	gagttggatgctggatggAGAGCGAAGACGTGGTATT
DA2-F	ggagtgagtacggtgtgcTTGTTCGATTAATGGCACCTTCAG
DA2-R	gagttggatgctggatggGTGTCATTGGCAATCTGATGCATG
NCED1-F	ggagtgagtacggtgtgcCGGCCTCGTCTACTTCAAC
NCED1-R	gagttggatgctggatggTACTTGAGGTACGGCTTCT
LPR2-F	ggagtgagtacggtgtgcCTCAGTGGCAGCCTGAGTACTTCG
LPR2-R	gagttggatgctggatggTGACGACGTCAAACGACTCGGACAC

Table S3. Heredity of genotypes from the T0 to T1 generations.

Target Gene	T0 Line	Genotype of T0 Lines	Bar Positive of T1 Plants	Genotypes of T1 Plants
TaPinb	1-22	-	132	9HetD:1ChiD
TaDA1	6	HetAHetB	3	5HomAChiB:1HetAHetB:2HetB:2HetAChiB:1HomAHetB:1HetAChiB
	14	HetAHetB	12	8HomAChiB:1HomAHetB:2BiaAChiB:1HetAChiB:2BiaAHetB:1HetAHetB:1 HomB:1HomAHetB:2BiaB:1HetB
	15	ChiAHetB	7	1HomAHomB:8BiaAChiB:14HomAChiB:1BiaABiaB:1ChiB
	17	HetAChiB	20	1BiaAHetB:9HomAChiB:2HomAHetB:3HetAChiB:3HetAHetB:1HomAHom B:2ChiB:1ChiAHetB:1HomB:2ChiB:1ChiAChiB
TaDA2	3	HetD	14	14HetD: 1HetAHetD
	5	HetD	12	1HomAHomBChiD:3HetD:4HetBHetD:
	58	BiaAHomBHomD	19	1HomAHomBHomD:5HomAHomBBiaD:2HomAHomBChiD:1HomABiaBC hiD:4BiaBBiaD:1HomABiaD:2HomBBiaD:1BiaABiaD:1HomAChiD
	60	BiaABiaB	19	1HetAHetBHetD:3HomAChiD:2HetAChiD:1HomABiaD:1HetBHetD:1BiaD: 5HetD
TaNCED1	53	HetAHetBHetD	9	3HetD: 2HetAHetD: 2HetAHetBHetD
	84	HetAHetB	18	8HetA: 6HetAHetBHetD
	98	HetAHetB	9	6HetA: 10HetAHetBHetD
	107	HetAHetB	5	7HetA: 4HetAHetBHetD
TaLPR2	1	ChiABiaD	16	1BiaAHomBBiaD:5ChiAHomD:5BiaABiaD:1BiaAChiD:6HomAChiD:2Hom ABiaD:2BiaAHomD
	54	HomD	18	9HomA:3BiaA:1HetAHomD:1BiaABiaD:4BiaAHomD:4HomAHomD:1HetA HetD
	57	HomABiaD	17	4HomAHomD:5HomABiaD:4HetA:1HomA
	59	ChiAChiD	21	1BiaAHomBHomD:3HetA:2HomB:2HomAHomD:1HetAHomD:1HetABiaD :1ChiD:1BiaA:1HetAHetD:1HetAHomB

Hom, homozygous; Het, heterozygous; chi, chimeras.

Table S4. Heredity of genotypes from the T1 to T2 generations.

Target Gene	T1 Line	Genotype of T1 Lines	Bar Detection(+/-) of T1 Plants	Genotype of T2 Plants
TaPinb	47	HetD	+	1HetD
	30	HetD	+	7HomD:9HetD
	34	HetD	+	1HomD:1HetD
	44	HetD	+	1HomD:1HetD
	48	HetD	+	1HetD
TaDA1	6-3	HetAHetB	+	9HomAChiB:3HetAHetB:3HetAChiB: 2HetB
	14-1	HomB	+	11HomAChiB:1HomAHomB:1HomAHetB:1HomABiaB:3ChiB:2BiaB
	14-15	HomAChiB	+	18HomAChiB:1BiaAChiB
	14-6	HomAChiB	-	16HomAChiB:2HomAHetB
	15-7	BiaABiaB	+	5HomAChiB:10BiaAChiB
	15-8	HomAChiB	-	11HomAChiB:3HomAHomB:1HomB
	17-4	HetB	+	1HomAHetB:5HomAChiB:1HomABiaB:1BiaABiaB:4BiaAChiB:3ChiB:1HetB
TaDA2	3-13	HetAHetD	+	12HetD
	3-12	HetD	-	13HetD
	5-13	HomAHomBChiD	+	16HetBHetD
	58-6	HomAHomBChiD	+	6BiaABiaD:12BiaAChiD:2BiaAHetD:1BiaA
	58-10	HomAHomBChiD	+	9HomAHomBChiD:4BiaAHomBBiaD:3HomAChiD
	58-18	HomAHomBHomD	+	1HomAHomBHomD:1HomAHomBChiD:1BiaAHomBHomD:1HomAHomBBiaD:1HomAHomBHetD:2HomAHomBHetD:2HomAHetBChiD:2HomAHetBHetD:1BiaAHetBHetD
	60-3	HetD	-	1HetD
TaNCED1	53-1	HetD	+	2ChiAChiBChiD:1ChiAHetBHetD:3HetAHetBHetD:2HetBHetD:3HetAHetD:5HetD
	53-6	HetAHetBChiD	+	8ChiAChiBChiD:12HetAHetBHetD
	53-10	HetAHetBHetD	+	15HetAHetBHetD
	84-6	HetA	+	4HetAHetBHetD:6HetA
	84-10	HetAHetBHetD	+	14HetAHetBHetD
	98-3	HetAHetBHetD	-	8HetAHetBHetD
	98-9	HetAHetBHetD	+	8HetAHetBHetD:1ChiAHetBChiD:7HetAHetD

	98-12	HetA	+	5HetAHetBHetD:2HetAHetD:7HetA
	98-13	ChiAHetD	+	4ChiAHetBHetD:3HetAHetBHetD:3ChiAHetD:1HetAHetD
	107-2	HetA	+	1HetAHetBHetD: 2HetA
TaLPR2	1-13	BiaAHomBBiaD	+	2BiaAHomBHomD:1BiaAHomBBiaD:3BiaABiaD:5BiaAHomD:3HomABiaD:5 HomAHomD:1ChiAHetD
	54-23	HetAHetD	-	13HomD:7HetD
	57-1	HetA	+	1HomA:1HetAHetD:1HomAHetD:8HetA:2ChiA:
	59-4	BiaA	+	1HomAHomBHomD:4HomAHomD:1ChiAChiD:1BiaAHomD:1BiaA:4ChiA HetD:2HomA:2HomD
	59-11	HomB	+	2HetAHomBHomD:3HetAHomBHetD:5HomBHomD:7HomBHetD:1HomAB iaD:1HomAChiD:1HomB
	59-14	BiaAHomBHomD	+	2HomAHomBHomD:7HomAHomB:2HomAHomD:1HomABiaD:1HomB:2H omD
	59-16	HetAHomD	+	12HomA:4HomAHomD:1BiaA:1HetA

Hom, homozygous; Het, heterozygous; chi, chimeras.

Table S5. Mutations in the putative CRISPR/Cas9 off-target sites.

Target	Genome Location	Sequence	No.of Mismatching Bases	Primers	No. Of Plants Detected	No. Of Plants with Mutations
Pinb-Off target	chr3B_591364632..591365116	CCGCATGGAAGCGGCCGAAGCTGAG	3	F:5′-GTCGTGCCATATCGTTGGCAA-3′<br>R:5′-GGCTCTTGACTTCCTCTTG-3′	24	0
DA1-Off target	chr3B_404628830..404629312	GAAATGAGATGATGCATGCATGG	3	F:5′-GCTATTGTCCGTATCATTGGC-3′<br>R:5′-GTTCCTTATTCATACGATCCAC-3′	24	0
DA2-Off target	chr7B_126807007..126807487	GTCTTACTTG-GACTCGAG-CGG	2	F:5′-GCACCAGCAGTTAAGTAGA-3′<br>R:5′-AACGAGGAAGAGTAGAACAC-3′	24	0
NCED1-Off Target1	chr1A_7311594..7312076	CCGGCGACCTCTCCGCGCTCTGC	3	F:5′-GAGACGAAGCTCATCGAGGA-3′<br>R:5′-TGCCGAAGGCGTAGGCGTTG-3′	24	0
NCED1-Off Target2	chr2A_376194240..376194722	CCGGATAGCTCTTCGCGTTCAGC	3	F:5′-TCGGAGATGGCATCAATAC-3′<br>R:5′-TCAGGCAAGGCTAAGAAC-3′	24	0
LPR2-Off target	chr2A_387111290..387111770	GTGTT-CTTGT-GCTCGAGTGGG	3	F:5′-CCACCTTGCGGTGTAAAACCAT-3′<br>R:5′-CCTAGCCACCACCATAGAGA-3′	24	0
